# Trajectories of appetitive traits and dietary patterns from childhood to adolescence: findings from the Generation XXI birth cohort

**DOI:** 10.1007/s00394-026-03947-3

**Published:** 2026-03-24

**Authors:** Alexandra Costa, Milton Severo, Rita Pereira, Marion M. Hetherington, Carla Lopes, Andreia Oliveira

**Affiliations:** 1 EPIUnit ITR, Instituto de Saúde Pública da Universidade do Porto, Universidade do Porto, Rua das Taipas, n° 135, Porto, 4050-600 Portugal; 2 Instituto de Ciências Biomédicas Abel Salazar, Universidade do Porto, Rua de Jorge Viterbo Ferreira, 228, Porto, 4050-313 Portugal; 3 School of Psychology, University of Leeds, Woodhouse Lane, LS2 9JT Leeds, UK; 4 Faculdade de Medicina, Departamento de Ciências da Saúde Pública e Forenses, e Educação Médica, Universidade do Porto, Alameda Prof. Hernâni Monteiro, Porto, 4200-319 Portugal

**Keywords:** Appetite, Dietary patterns, Behavior, Cohort studies, Pediatric population

## Abstract

**Purpose:**

This study examined the association between appetitive trait profiles and dietary patterns from childhood to early adolescence.

**Methods:**

We included 5040 participants from the Generation XXI cohort (Porto, Portugal). Appetitive traits were assessed at ages 7, 10, and 13 with the validated Children’s Eating Behaviour Questionnaire. Six trajectory-based profiles were previously identified: “Smallest appetite”, “Small appetite but increasing”, “Small to moderate appetite”, “Moderate appetite”, “Increasing appetite”, and “Avid appetite”. A validated Food Frequency Questionnaire collected dietary intake at ages 4, 7, 10, and 13. Latent class analyses identified dietary patterns at each age and dietary patterns trajectories across childhood. Multinomial logistic regression assessed the association of appetitive trait profiles and dietary patterns.

**Results:**

Three dietary patterns (“Healthier”, “Lower Consumption”, and “Energy-Dense Foods” (EDF)) and two trajectories from 4 to 13 years (“Predominantly Healthier” and “Predominantly EDF”) were identified. Compared with the “Moderate appetite” profile, children in the “Small appetite but increasing” (characterised by food avoidance and high Desire to Drink) had 54% higher odds of the “Predominantly EDF” trajectory. Conversely, individuals in the “Small to moderate appetite” profile (lowest food approach and emotional eating, suggestive of better appetite regulation) and “Avid appetite” profile were less likely to follow the “Predominantly EDF” trajectory.

**Conclusions:**

Appetitive trait profiles are linked to dietary patterns from childhood to adolescence. Food avoidance combined with a high Desire to Drink predicted less healthy diets, while stronger food approach traits and a profile indicative of improved appetite regulation were linked to healthier dietary choices.

**Supplementary Information:**

The online version contains supplementary material available at 10.1007/s00394-026-03947-3.

## Introduction

Appetitive traits are stable tendencies toward food that emerge in early life, including eating in response to external food cues, sensitivity to satiety signals, and emotional eating [1]. These are commonly categorised into food approach traits (Food Responsiveness, Enjoyment of Food, Emotional Overeating, and Desire to Drink), which reflect an avid appetite and high interest in food, and food avoidance traits (Satiety Responsiveness, Slowness in Eating, Food Fussiness, and Emotional Undereating), which reflect a more selective attitude toward eating and a smaller appetite [2, 3].

Evidence suggests that the food approach traits Enjoyment of Food and Food Responsiveness are associated with higher overall food intake, including greater consumption of fruits and vegetables [2–5]. On the other hand, Desire to Drink, has been associated with higher intake of sugar-sweetened beverages and energy-dense foods, as well as lower overall diet quality [3, 5–7]. Food avoidance traits, particularly Food Fussiness, are generally linked to lower food intake and reduced consumption of fruits and vegetables [2, 3, 5, 8, 9]. Some studies also indicate that food avoidance traits are related to poorer diet quality and higher consumption of less healthy foods [5, 10–12]. For instance, Satiety Responsiveness has been associated with increased intake of snacks [2, 6], sweets [4], and energy-dense foods [3], although these findings remain inconsistent [5, 6, 13].

It is important to note, however, that most evidence relies on cross-sectional designs or studies with short follow-up periods, and therefore does not capture how associations unfold over time [14]. Additionally, most research evaluates isolated dietary components (e.g., specific food groups such as fruits or vegetables) or composite diet quality indices, which overlook the interactive and cumulative nature of dietary intake [15]. In contrast, dietary pattern analysis, using various food components and repeated measures over time, provides a more comprehensive picture of overall habitual dietary intake [16].

Similarly, appetitive traits co-occur and interact. The combination of appetitive traits within an individual characterises overall eating behaviours, resulting in different eating phenotypes across the population. Recent studies have adopted a person-centred approach to identify eating phenotypes based on the clustering of appetitive traits [17–19]. In Generation XXI, our previous work identified profiles of appetitive trait trajectories from ages 7 to 13, revealing subgroups with consistent food avoidance or food approach tendencies [19]. These profiles were associated with body mass index (BMI) and cardiometabolic health [19, 20], yet their relationship with food consumption remains unexplored. Understanding these links is essential to fully characterise these appetitive profiles, clarify their role in shaping dietary choices, and evaluate their potential as mediators of long-term health. To date, no study has investigated the association between trajectories of appetitive traits and diet within Generation XXI or other populations.

The present study aims to address these gaps by exploring the association between appetitive trait profiles and dietary patterns across childhood and early adolescence, using a unique population-based dataset that includes repeated dietary assessments from ages 4 to 13 and appetitive trait measures from ages 7 to 13.

## Material & methods

### Study population

Data were drawn from Generation XXI, a prospective population-based birth cohort study assembled in Porto, Portugal [21, 22]. Between April 2005 and August 2006, 8647 liveborn infants were enrolled in all the public maternity wards in the Porto Metropolitan Area. Follow-up evaluations were conducted at ages 4, 7, 10, and 13 years, with participation proportions of 86%, 80%, 76%, and 54%, respectively (the latter interrupted due to the COVID-19 pandemic).

For the main analyses, we included 5040 participants who had both appetitive trait trajectory profiles and food consumption data from at least two evaluations between ages 4 and 13. Trajectories were based on at least two measurements of the Children’s Eating Behaviour Questionnaire (CEBQ) at ages 7, 10, and/or 13 years (70% with all three). In multiple births, one child per family was randomly selected. All participants with data on these profiles also had available food consumption data (Supplemental Material 1). Compared with the rest of the cohort (*n* = 3607), mothers in this sample were, on average, slightly older (29.9 ± 5.1 vs. 27.7 ± 6.0 years, *p* < 0.001) and more educated (11.3 ± 4.2 vs. 9.2 ± 3.9 years, *p* < 0.001). Effect sizes were not large (Cohen’s d = 0.39 and 0.50, respectively), suggesting that significant differences were likely attributable to the large sample size rather than systematic differences between the two groups [23].

### Ethical approval

All phases of the study complied with the Ethical Principles for Medical Research Involving Human Subjects expressed in the Declaration of Helsinki. The baseline and follow-up evaluations were approved by the University of Porto Medical School/ S. João Hospital Centre Ethics Committee, except the 13-year follow-up, which was approved by the ISPUP Ethics Committee. At baseline and follow-up evaluations, all procedures were explained to participants, and informed consent was signed by one of the parents or legal guardians (at 13 years, it was also signed by the participants). The baseline evaluation was additionally approved by the Data Protection National Commission, and the study follows the present EU General Data Protection Regulation under close supervision of the Data Protection Office of ISPUP.

### Data collection

Data were collected through face-to-face interviews with (caregivers/ and or children) conducted by trained researchers or by self-administered questionnaires. At baseline assessment, the child’s sex, maternal age, education, height and weight before pregnancy, and monthly disposable household income were gathered. Birthweight was retrieved from medical records. Children’s anthropometrics were measured at each follow-up, following standardised procedures. Age- and sex-specific BMI z-scores were then calculated based on WHO criteria for sample characterisation [24]. At age 4, parents reported any scheduled or regular sports their children participated in, along with the average time spent per week on each activity. The intensity of physical activity was quantified in metabolic equivalents of task (METs), with MET values for each activity derived from the Youth Compendium of Physical Activities [25]. Only moderate-to-vigorous physical activity (≥ 3 METs) was considered, and total weekly duration was subsequently calculated.

#### Appetitive traits

Appetitive traits were assessed with the CEBQ, 35-item parent-administered instrument measuring eight subscales (food approach traits: Food Responsiveness, Enjoyment of Food, Emotional Overeating, Desire to Drink; food avoidance traits: Satiety Responsiveness, Slowness in Eating, Emotional Undereating, and Food Fussiness) [26]. Profiles of appetitive trait trajectories from 7 to 13 years of age were estimated using a person-centred approach; a detailed description can be found elsewhere [19]. Briefly, linear mixed-effect models were run to estimate the individual trajectory of appetitive traits by considering the random slope and intercept. The extracted random effects were then used in Gaussian mixture models fitted with the Expectation–Maximisation (EM) algorithm to identify participants with similar longitudinal patterns. Six appetitive trait trajectory profiles were identified: “Smallest appetite” (highest food avoidance and low food approach), “Small appetite but increasing” (decreasing of both food avoidance and Desire to Drink but with the highest levels of Desire to Drink of all profile), “Small to moderate appetite” (lowest food approach and emotional eating), “Moderate appetite” (scores for each trait close to the sample average), “Increasing appetite” (increasing food approach and decreasing food avoidance over time), and “Avid appetite” (highest food approach and lowest food avoidance) (Supplemental Material 2).

#### Food consumption

Food consumption was assessed with a qualitative Food Frequency Questionnaire (FFQ) applied by a trained staff member to the parents or the child’s primary caregiver at 4, 7, and 10 years, capturing food consumption over the last 6 months. At 13 years, it was applied by a trained staff member directly to the adolescent, capturing food consumption in the previous 12 months. The total number of items used in the FFQ was 35, 38, 41 and 39 at ages 4, 7, 10 and 13, respectively; the number of items varied due to adjustments to improve the instrument and age-appropriate changes. A nine-point scale captured frequency from “never” to” ≥4 times/day”. The FFQ was tested and calibrated in a sub-sample from Generation XXI using 3-day food diaries and nutrient biomarkers [27]. At each age, mean daily consumption (g/day) was estimated using a z-score calibration method, in which standardised FFQ frequencies were adjusted using the mean and standard deviation of intake (g/day) derived from 3-day food diaries (2 weekdays and 1 weekend day), as detailed elsewhere [27].

### Statistical analysis

Food consumption data from ages 4, 7, 10, and 13 years were transformed into a long-format dataset. Participants with more than 10 missing items at a given age were excluded. Food items were harmonised across ages, and items asked only at specific time points were excluded (*n* = 36). Then rice, pasta, and potatoes were additionally excluded because of limited variability in consumption, which reduced their ability to differentiate individuals or contribute meaningfully to dietary characterisation. This process yielded a final dataset containing 32 food items. For each food item, daily intake (grams/day) was categorised into three groups based on quintiles: lower (1st quintile), intermediate (2nd − 4th quintiles), and higher consumption (5th quintile) (Supplemental Material 3). The intermediate and higher categories were combined for fish/seafood, eggs, and soup due to identical cut-offs.

To identify dietary patterns at each age, we performed a time-invariant Latent Class Analysis (LCA) using the poLCA package [28]. We chose this method because our data consisted of categorical, asymmetrically distributed responses, which are well-suited to LCA. The analysis included all 5247 participants with dietary data from at least two time points. We tested models with two to eight classes. The ideal number of classes was determined based on the Akaike Information Criterion (AIC), Bayesian Information Criterion (BIC), scree plots, and interpretability. Each participant was then assigned to the class with the highest posterior probability of membership at each age. Then, following the same method, we derived dietary pattern trajectories from ages 4 to 13 years by performing a second LCA on participants’ class assignments across all ages (i.e., their dietary pattern at each age). This approach grouped children with similar patterns over time and enabled the identification of distinct trajectories.

Associations between appetitive trait profiles and dietary patterns were analysed using multinomial logistic regression, with odds ratios (ORs) and 95% confidence intervals (CIs). We ran both unadjusted (crude) and adjusted models (child’s sex, physical activity at age 4 (hours/week in moderate-to-vigorous physical activity), mothers’ education, age, and household income). We tested other factors such as maternal BMI, birth weight, and smoking during pregnancy, but associations did not change, so we decided to adopt a more parsimonious model. Potential interactions between children’s sex and appetitive trait profiles were tested but removed from final models as they were non-significant. Missing data for our covariates were handled using multiple imputations with the Multivariate Imputation by Chained Equations (MICE) package, assuming the data were missing at random. The percentage of missing data was low (3% for income, 8% for physical activity, and close to zero for others). Multicollinearity was assessed using tolerance and the variance inflation factor (VIF), with no evidence of collinearity. To test the robustness of our findings, we performed a sensitivity analysis, including only participants with plausible dietary reports. Misreporting was estimated by calculating the ratio between the reported total energy intake (TEI) and the estimated energy requirement (EER) [29, 30]. The EER was calculated using validated sex- and age-specific equations, considering the physical activity category (low active), exact age, and objectively measured weight and height [31]. Participants were categorised as plausible reporters, under-reporters, or over-reporters of TEI using the ± 1 SD cut-off for the ratio TEI/EER [29, 30].

Statistical analyses were performed using R 4.3.0. and the level of significance was set a priori at *p* < 0.05.

## Results

### Characteristics of the sample

Table 1 presents participants’ characteristics. The most common appetitive trait trajectory profile was the “Moderate appetite” (29.1%), followed by the “Small to moderate appetite” (26.1%), while the least frequent was the “Small appetite but increasing” (8.1%). The two most extreme profiles, “Avid appetite” and “Smallest appetite,” were observed in 11.8% and 10.4% of participants, respectively.

**Table 1:** Characteristics of study participants from the Generation XXI birth cohort (*n* = 5040)

Family characteristics at child’s birth	
Maternal age (y), mean (SD)	29.9 (5.1)
Maternal education (y), mean (SD)	11.3 (4.3)
Maternal pre-pregnancy BMI, (kg/m²) mean (SD)	23.9 (4.2)
Household income (euros), n (%)	
Low (≤ 1000)	1503 (31.3)
Intermediate (1001–2000)	2127 (44.3)
High (≥ 2001)	1169 (24.4)
Smoking during pregnancy, n (%)	
Ever smoked	971 (19.6)
Never smoked	3992 (80.5)
Child characteristics	
Sex, n (%)	
Female	2455 (48.7)
Male	2585 (51.3)
Birth weight (kg), mean (SD)	3.2 (0.5)
BMI z-scores 4y, mean (SD)	0.6 (1.1)
BMI z-scores at 7y, mean (SD)	0.7 (1.2)
BMI z-scores at 10y, mean (SD)	0.7 (1.2)
BMI z-scores at 13y, mean (SD)	0.4 (1.2)
Physical activity (moderate-to-vigorous) per week at 4y (h), mean (SD)	1.1 (1.1)
Appetitive trait trajectory profile ^a^, n (%)	
Moderate appetite	1462 (29.1)
Small appetite but increasing	408 (8.1)
Small to moderate appetite	1312 (26.0)
Avid appetite	597 (11.8)
Increasing appetite	739 (14.7)
Smallest appetite	522 (10.4)

y: years old. BMI z-scores: body mass index z-scores. ^a^Appetitive trait profiles were driven with data from the Children’s Eating Behaviour Questionnaire at 7,10, and 13 years of age [19]. n varies due to missing values

### Dietary patterns

**Fig. 1:**
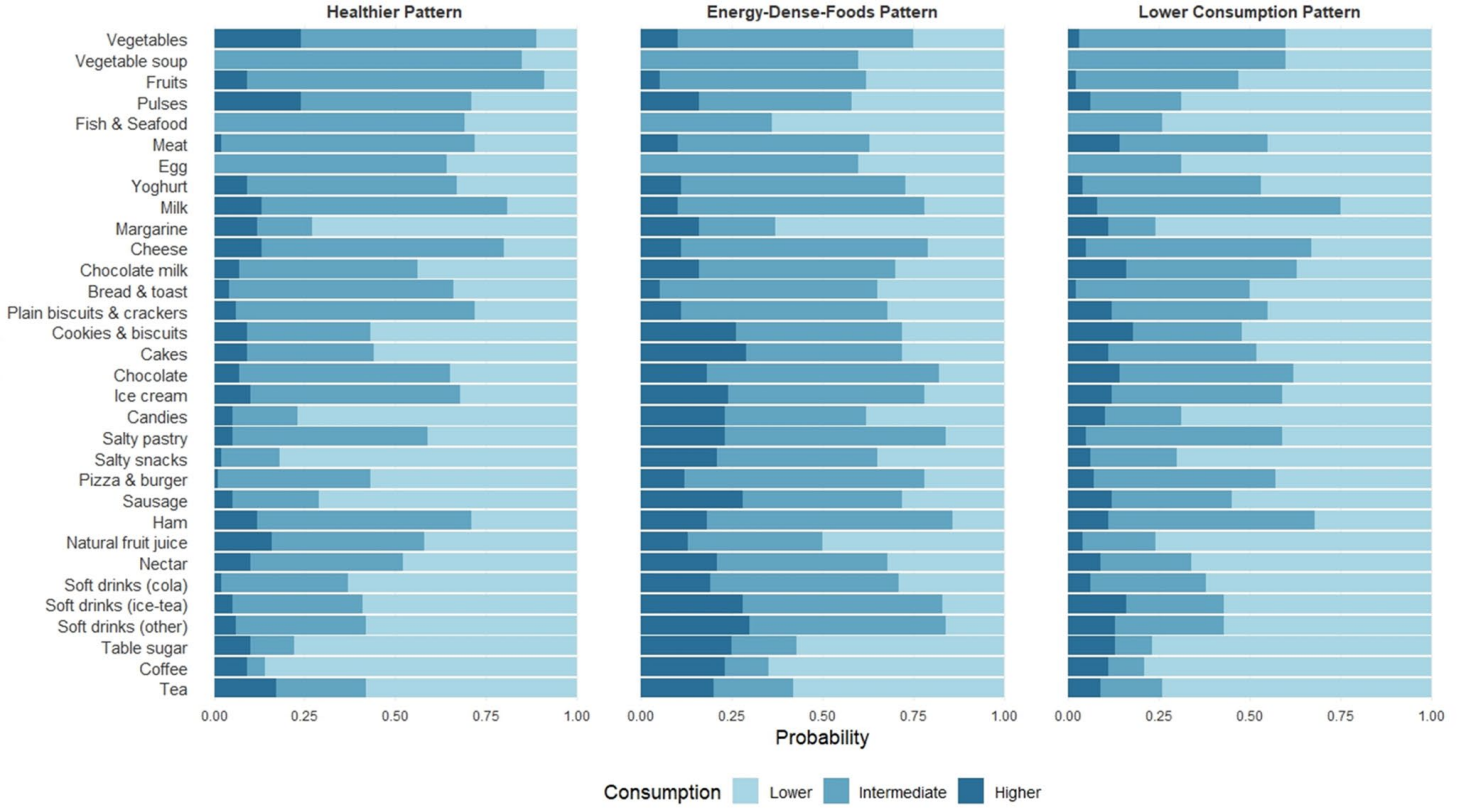
Conditional probability by food item for each dietary pattern. Latent class analysis was run using dietary data from ages 4, 7, 10, and 13 years (*n* = 5247). Daily consumption was categorised into three groups: lower (1st quintile), intermediate (2nd − 4th quintiles), and higher consumption (5th quintile). For fish/seafood, eggs, and soup, the intermediate and higher categories were combined

To derive dietary patterns, the 3-class model was selected, presenting the best balance between statistical fit and interpretability (AIC = 1,111,752; BIC = 1,113,226) with balanced class sizes, high classification quality (posterior probabilities: 0.85–0.94), and three distinguishable dietary patterns. Although a 4‐class model had slightly better fit indices (AIC = 1,107,540; BIC = 1,109,507), it was rejected because the extra class was relatively small (17%) and overlapped with two existing classes, making it less interpretable and lowering its overall classification quality. The scree plot for the BIC indicated that improvements in model fit were minimal beyond the 3‐class solution. Class 1 (“Healthier”) showed the highest probability of consuming nutrient-dense foods, including vegetables, vegetable soup, pulses, fruits, and fish/seafood, and the lowest probability of consuming energy-dense foods, including soft drinks, processed meats, salty snacks, and sweets. Class 2 (“Energy‐Dense Foods” (EDF)) displayed the opposite combination, with higher probabilities of consuming energy-dense foods (soft drinks, processed meats, salty snacks, pastries, and sweets, among others) and lower probabilities of consuming nutrient‐dense foods (such as fruits, vegetables, and pulses). Class 3 (“Lower Consumption”) had a lower consumption of energy-dense foods than Class 2 but slightly higher than Class 1, alongside a lower consumption of fish/seafood, fruit, pulses, and vegetables. Overall, this pattern had a greater likelihood of being in the lowest consumption category across several food items. Conditional item response probabilities are presented in Fig. 1 and Supplemental Material 4.

Dietary pattern prevalence shifted with age (Table 2). The “Lower Consumption” pattern declined progressively, being the most common at age 4 and least prevalent after (51.5%, 30.0%, 18.2%, 21.1%, respectively at ages 4, 7, 10 and 13). The “Healthier” pattern increased from age 4 to age 10 (20.7%, 31.7%, 52.0%) and then declined at age 13 (33.7%). The “EDF” pattern fluctuated but became the most prevalent by age 13 (27.8%, 38.3%, 29.8%, 45.2%).

**Table 2:** Distribution of dietary patterns (at each age and longitudinal trajectories) by appetitive trait trajectory profiles from ages 7 to 13

Dietary patterns at each age	Appetitive trait trajectory profiles from ages 7 to 13					
	Smallest appetite	Small appetite but increasing	Small to moderate appetite	Moderate appetite	Increasing appetite	Avid appetite
	n (%)	n (%)	n (%)	n (%)	n (%)	n (%)
*Age 4*						
Healthier	109 (23.1)	53 (14.6)	265 (23.1)	272 (21.6)	107 (17.0)	103 (20.2)
Energy-Dense Foods	133 (28.2)	123 (33.9)	243 (21.2)	365 (29.0)	198 (31.4)	155 (30.3)
Lower Consumption	230 (48.7)	187 (51.5)	639 (55.7)	621 (49.4)	325 (51.6)	253 (49.5)
*Age 7*						
Healthier	155 (30.6)	82 (20.6)	464 (36.3)	434 (31.2)	203 (28.1)	205 (35.7)
Energy-Dense Foods	165 (32.6)	193 (48.5)	386 (30.2)	601 (43.2)	306 (42.3)	213 (37.1)
Lower Consumption	186 (36.8)	123 (30.9)	427 (33.4)	354 (25.5)	214 (29.6)	156 (27.2)
*Age 10*						
Healthier	269 (52.8)	163 (40.6)	736 (56.7)	748 (51.4)	332 (46.7)	324 (56.1)
Energy-Dense Foods	136 (26.7)	150 (37.4)	300 (23.1)	482 (33.1)	239 (33.6)	169 (29.2)
Lower Consumption	104 (20.4)	88 (21.9)	261 (20.1)	224 (15.4)	140 (19.7)	85 (14.7)
*Age 13*						
Healthier	145 (31.7)	69 (20.9)	445 (37.5)	379 (33.4)	219 (31.2)	195 (38.9)
Energy-Dense Foods	205 (44.8)	176 (53.3)	507 (42.7)	548 (48.2)	323 (46.1)	190 (37.9)
Lower Consumption	108 (23.6)	85 (25.8)	234 (19.7)	209 (18.4)	159 (22.7)	116 (23.2)
*Trajectories of dietary patterns (4 to 13)*						
Predominantly Healthier	235 (44.2)	121 (29.0)	622 (41.6)	673 (50.1)	285 (47.0)	273 (36.6)
Predominantly Energy-Dense Foods	297 (55.8)	296 (71.0)	873 (58.4)	670 (49.9)	321 (53.0)	473 (63.4)

Appetitive trait profiles were driven with data from the Children’s Eating Behaviour Questionnaire at 7,10, and 13 years of age [19]. n varies due to missing values

**Fig. 2:**
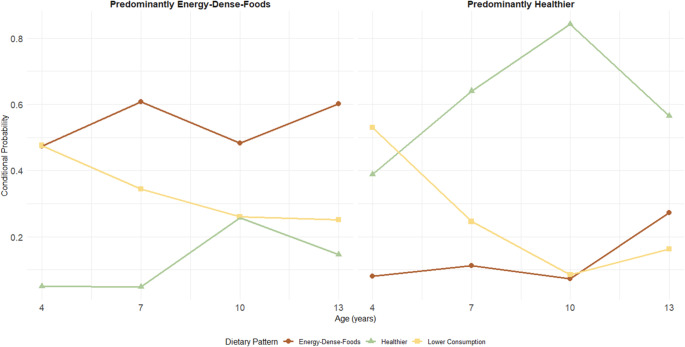
Trajectories of dietary patterns across ages 4,7,10, and 13. Trajectories identified with latent class analysis (*n* = 5247), based on the class membership of each dietary pattern previously identified: Healthier, Energy-Dense Foods, and Lower Consumption

Regarding dietary pattern trajectories, the best solution was a 2-class model, having the lowest BIC and AIC (AIC = 52,558; BIC = 52,755). Class 1 (“Predominantly Healthier”) (47.0%) included participants who were most often classified in the “Healthier” or “Lower Consumption” patterns across childhood and adolescence, with low probability of being in the “EDF” pattern. Class 2 (“Predominantly EDF”) (43.0%) was characterised by consistently higher probabilities of being in the “EDF” and “Lower Consumption” patterns throughout age (Fig. 2).

### Trajectories of appetitive trait profiles and dietary patterns

**Fig. 3:**
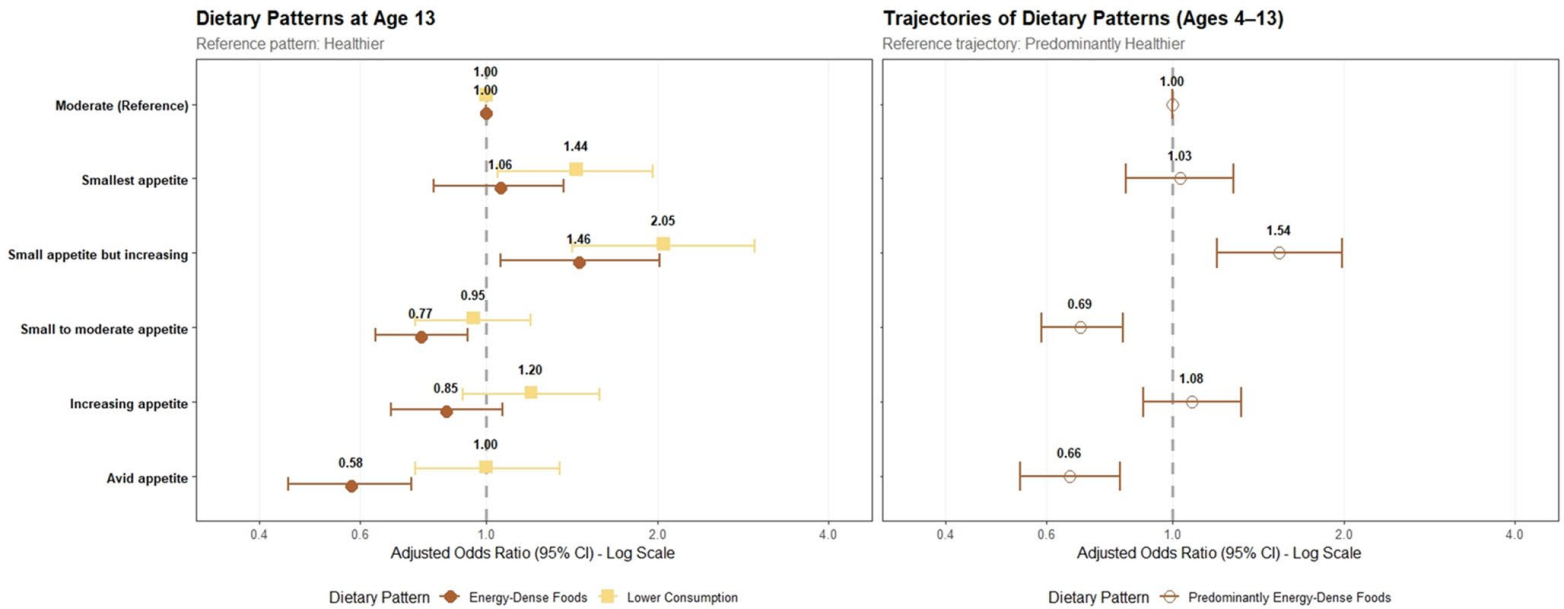
Associations between appetitive trait trajectory profiles and two outcomes: the dietary patterns at the older age (age 13) and using the longitudinal trajectories from 4 to 13 years. OR: Odds ratio. CI: confidence interval; ref: reference category. ^a^ Dietary Patterns identified with latent class analysis, using dietary data collected at 4, 7, 10, and 13 years of age. Appetitive trait profiles were driven with data from the Children’s Eating Behaviour Questionnaire at 7,10, and 13 years of age [19]. Models adjusted for child’s sex, physical activity at age 4, mothers’ education, age, and household income. N is 4232 at age 13, and 5040 for the trajectories

At age 13, compared with the “Moderate appetite”, children in the “Small appetite but increasing” profile had higher odds of following the “EDF” pattern and the “Lower Consumption” pattern than the “Healthier” (Fig. 3 and Supplementary Material 5). The “Smallest appetite” profile was associated with greater odds of the “Lower Consumption” pattern. On the other hand, children in the “Small to moderate appetite” and the “Avid appetite” had lower odds of following the “EDF” pattern. Sensitivity analysis (Supplemental Material 6), excluding individuals with potential dietary misreporting, yielded broadly consistent results.

Considering the longitudinal dietary trajectories, children with a “Small appetite but increasing” had 54% higher odds of following the “Predominantly EDF” trajectory compared with the “Predominantly Healthier”. Conversely, the “Small to moderate appetite” and “Avid appetite” profiles were associated with about 30% lower odds of following the “Predominantly EDF” trajectory. These results were consistent in sensitivity analyses.

## Discussion

This study examined the relationship between appetitive trait profiles from ages 7 to 13 years and dietary patterns from childhood to adolescence. We identified three dietary patterns (“Healthier”, “EDF,” and “Lower Consumption”) and two longitudinal trajectories (“Predominantly Healthier” and “Predominantly EDF”). Profiles characterised by high food avoidance, particularly when combined with a strong Desire to Drink (i.e., “Small appetite but increasing” profile), were associated with less healthy dietary patterns. In contrast, children with a “Small to moderate appetite” and “Avid appetite” profiles were more likely to follow a healthier dietary pattern, including more nutrient-dense foods.

The dietary patterns we found differed mainly in the balance between nutrient-dense foods (e.g., fruits, vegetables, pulses, fish) and energy-dense foods (e.g., soft drinks, sweets, snacks, fast food); a contrast frequently reported in the literature on data-driven dietary patterns, including previous analyses within our cohort at specific ages [32–35]. Our approach captures dietary patterns observed across childhood rather than focusing on age-specific consumption nuances, allowing the identification of groups of children who shared similar fluctuations in dietary patterns over time (at 4, 7, 10 and 13 years of age). Overall, this analysis provides a comprehensive overview of how dietary choices cluster and evolve during childhood.

The “Small to moderate appetite”, characterised by the lowest food approach and emotional eating from ages 7 to 13, was associated with lower odds of following the “EDF” pattern at age 13 and the “Predominantly EDF” trajectory. Despite their low drive to eat and low interest in food, children in this profile were more likely to follow a pattern with greater intake of fruits and vegetables and lower intake of energy-dense foods, suggesting that improved self-regulation of eating (i.e., responding to internal cues of hunger and satiety and having less emotionally driven consumption) may support healthier food choices. The low emotional eating scores reveal that these children do not seek food out for comfort when experiencing negative emotions. Our person-centred approach, which considers combinations of traits rather than traits in isolation, allowed us to uncover this more nuanced relationship. Overall, low food approach still includes intake of nutrient dense foods, also this profile has an average (instead of higher) food fussiness scores. It is also important to note that the “Small to moderate appetite” profile has previously been associated with higher maternal age and feeding practices such as portion control, selection of food types, and monitoring of the child’s consumption [6]. These parental influences may contribute to a healthier food environment, reinforcing self-regulated, balanced eating and, thus, better food choices.

The “Avid appetite” profile was also associated with a healthier dietary pattern. This finding is consistent with studies linking an avid appetite to a greater dietary variety and acceptance of nutrient-dense foods [5, 14, 36]. The observed associations may be mainly driven by two specific food approach traits: Food Responsiveness and Enjoyment of Food, which, when measured at age 7 in our cohort, predicted higher consumption of fruits and vegetables and lower consumption of soft drinks at age 10 [3]. However, these associations are complex and seemingly paradoxical. While this profile seems to reflect higher dietary quality, it has been associated with higher BMI and worse cardiometabolic health [19, 20].

This paradox likely reflects a measurement limitation. The questions included in the CEBQ that define the eating trait cluster assess how children enjoy eating and respond to food, without mentioning which specific types of foods. Therefore, one hypothesis is that children in this profile may consume larger quantities of all foods, including healthy ones, leading to higher energy intake relative to energy requirements and, ultimately, higher weight and metabolic risk. Also, larger quantities may not be fully captured by the FFQ, as it is designed primarily to assess the types and frequencies of foods consumed rather than the exact amounts consumed. Another possibility is reverse causality, as children or parents may adapt dietary behaviours in response to weight concerns or to manage a voracious appetite. Attenuation of associations in sensitivity analyses (becoming non-significant in the crude model) may also suggest some susceptibility to social desirability bias in dietary reporting. Underreporting was higher in this profile than in others, consistent with evidence that individuals with higher body weight tend to underreport intake, whereas leaner individuals may overreport [37]. Lastly, as not all avid eaters followed a healthier dietary pattern, for those with pronounced food approach tendencies, the food environment is probably a key nudging factor. However, when exposed to healthy food options, these children are less selective (fussy) and may be prone to accept nutrient dense foods, driving their diet toward healthier patterns in response to their overall greater appetite. Conversely, the same traits might predispose them to greater intake of less nutritious foods in more permissive environments. Future research should test whether the food environment moderates these associations.

In contrast, profiles dominated by food avoidance traits were associated with less healthy dietary patterns. The “Small appetite but increasing” profile —characterised by high Food Fussiness, the highest Desire to Drink, and low food approach traits — was more likely to follow the “EDF” and “Lower Consumption” patterns at age 13 and the “Predominantly EDF” trajectory. Previous studies have reported associations between food avoidance traits, particularly Food Fussiness, with lower adherence to healthy dietary patterns [5, 36, 38], reduced intake of fruits and vegetables [4, 5, 8], and greater consumption of energy-dense [3, 5, 7] and ultra-processed foods [11]. The prominent Desire to Drink trait may be especially relevant, as soft drink consumption strongly characterises the “EDF” pattern. Desire to Drink has been associated with higher intake of soft drinks [5–7], including in a study from our cohort [3], which further demonstrated that higher scores on this trait at age 7 were linked to lower intake of vegetables and fish, and higher intake of energy-dense foods at age 10. A recent study of children aged 3 to 6 also linked higher Desire to Drink with two dietary patterns: one characterised by processed meats and snacks, and another high in sugar and sweetened beverages [5].

The “Smallest appetite” profile, marked by the highest overall food avoidance (i.e., children with very low interest and liking of food), was only associated with the “Lower Consumption” dietary pattern — characterised by lower fruits, vegetables, pulses, and fish consumption, but more intermediate intake of energy dense-foods. This suggests that extreme food avoidance may restrict dietary variety through greater selectivity without necessarily promoting higher consumption of energy-dense foods.

A major strength of this study is the use of a large, population-based prospective cohort with repeated measures of dietary intake and appetitive traits from childhood through adolescence. This longitudinal design allowed us to identify dietary patterns and trajectories spanning nearly a decade, providing a more comprehensive picture of diet dynamics than single-time-point analyses. The person-centred analytic approach is also a strength, capturing heterogeneity in children’s behaviours and identifying subgroups that reflect meaningful combinations of food consumption and appetitive traits, rather than relying on isolated variables. This is among the first studies to examine how combinations of appetitive traits predict long-term dietary patterns.

Some limitations should also be acknowledged. Dietary intake was assessed through parent-reported FFQs in childhood and self-reported FFQs in adolescence, which are subject to recall and social desirability bias, particularly the over-reporting of healthy foods and under-reporting of energy-dense ones. In addition, differences between parent-reported and adolescent self-reported data at age 13 may contribute to reporting bias. Nonetheless, the FFQ has been calibrated against 3-day food records in this cohort, and portion sizes were carefully adjusted for each age group; we also conducted sensitivity analyses excluding individuals with potential misreport [27]. Regarding these sensitivity analyses, associations had the same direction; the magnitude decreased, as expected, considering the smaller sample size. The CEBQ, also based on parental report, is a reliable and validated tool, including against laboratory-based measures, but may still be prone to reporting bias [39, 40]. Additionally, using data-driven approaches has some constraints as they are partly sample-specific, and the number or nature of classes identified may differ across populations, potentially limiting generalizability. The labelling of classes involves some degree of interpretation, even though we considered widely accepted evidence regarding nutrient-dense versus energy-dense foods. Lastly, we did not have data on appetitive traits at age 4; thus, trajectory profiles were defined from age 7 onward. However, we included dietary data from age 4 to provide the most complete characterisation of dietary intake across childhood. This decision was supported by evidence suggesting a moderate degree of stability in appetitive traits [19, 41]. This lack of data remains a limitation, not being able to assure the precedence of the appetitive traits in relation to diet and raises the possibility of a reverse causality bias.

## Conclusions

Our study proposes a clear link between developmental profiles of appetitive traits (ages 7 to 13) and dietary patterns throughout childhood and adolescence (ages 4 to 13). Profiles marked by higher food avoidance, especially when coupled with a strong Desire to Drink, were consistently associated with less healthy dietary patterns. Conversely, children whose profiles reflected less severe food avoidance with an overall better appetite self-regulation, as well as those with a more avid appetite, had a healthier pattern. Early identification of these appetitive tendencies could aid targeted interventions to foster healthier eating habits. Children with smaller appetite who are selective in eating, who reject certain foods and have a high drive to consume soft drinks may require tailored strategies to improve diet quality. On the other, children with an avid appetite, are prone to consuming amounts exceeding their energy needs, even with healthier dietary patterns. For these children, approaches that enhance satiety may be useful, such as offering a greater availability of low energy density, high nutrient-dense foods (e.g., fruits, vegetables and high fibre foods), which allows higher food intake while avoiding excess energy intake. The home food environment is a key modifiable factor, with potential to offset avidity of appetite with offering healthy, filling, nutrient dense foods.

## Supplementary Information

Below is the link to the electronic supplementary material.

Supplementary Material 1

## Data Availability

The data from Generation XXI are not publicly available due to privacy and ethical restrictions. The data can be made available for research proposals on request to the Generation XXI Executive Committee (generationxxi@ispup.up.pt). Further information about Generation XXI can be obtained via the Generation XXI website [www.geracao21.com] or by emailing generationxxi@ispup.up.pt. Codebook and analytic code will be made available upon request.
