# Supplementary material for: Trajectories of appetitive traits and dietary patterns from childhood to adolescence: findings from the Generation XXI birth cohort
Source: Eur J Nutr. 2026 Mar 24;65(3):105. doi: 10.1007/s00394-026-03947-3 (PMC13013234; doi:10.1007/s00394-026-03947-3)
Supplement: Supplementary file 1 — Supplementary Material 1 [file 394_2026_3947_MOESM1_ESM.docx]

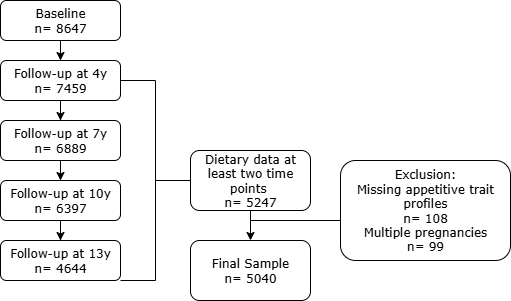


Supplemental Material 1. Flowchart of participant’s selection


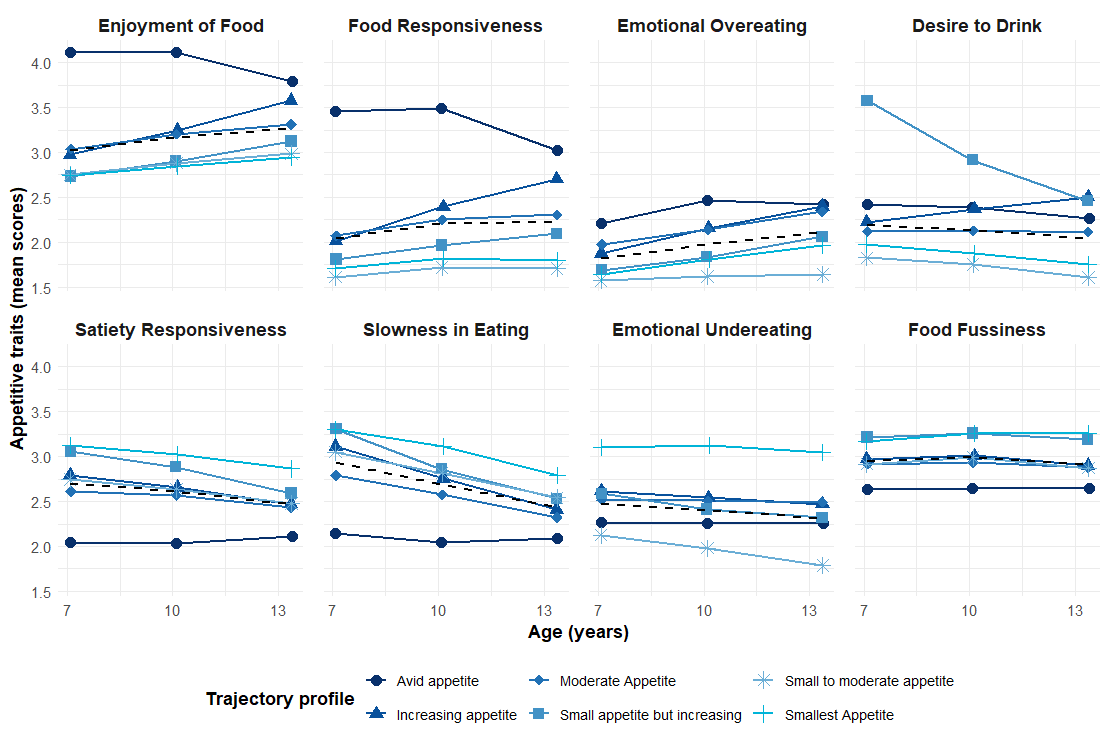
Supplemental Material 2. Mean scores for the eight Children's Eating Behaviour Questionnaire subscales according to the different appetitive trait trajectory profiles (n=5040).

Appetitive trait profiles were driven with data from Children’s Eating Behaviour Questionnaire at 7,10, and 13 years of age [1].

Supplemental Material 3. Cut-offs for daily consumption groups for the different food items.

|  | **Group/daily consumption in grams** | **Ages (years old)** | | | |
| --- | --- | --- | --- | --- | --- |
| **Food Item** |  | **4** | **7** | **10** | **13** |
| Bread & toast | Lower (P20)^a^ | 23 | 24 | 40 | 25 |
|  | Higher (P80) | 68 | 77 | 88 | 98 |
| Plain biscuits & crackers | Lower (P20) | 3 | 2 | 0 | 0 |
|  | Higher (P80) | 14 | 19 | 28 | 19 |
| Cookies & biscuits | Lower (P20) | 0 | 0 | 0 | 0 |
|  | Higher (P80) | 10 | 15 | 33 | 27 |
| Cakes | Lower (P20) | 0 | 7 | 8 | 17 |
|  | Higher (P80) | 10 | 15 | 24 | 28 |
| Chocolate | Lower (P20) | 0 | 1 | 0 | 0 |
|  | Higher (P80) | 4 | 4 | 16 | 15 |
| Ice cream | Lower (P20) | 0 | 0 | 0 | 0 |
|  | Higher (P80) | 9 | 8 | 11 | 20 |
| Candies | Lower (P20) | 0 | 0 | 0 | 0 |
|  | Higher (P80) | 2 | 3 | 3 | 3 |
| Salty pastry | Lower (P20) | 0 | 0 | 0 | 1 |
|  | Higher (P80) | 18 | 21 | 23 | 19 |
| Salty snacks | Lower (P20) | 0 | 0 | 0 | 0 |
|  | Higher (P80) | 1 | 2 | 4 | 2 |
| Pizza & burger | Lower (P20) | 0 | 0 | 3 | 0 |
|  | Higher (P80) | 6 | 24 | 44 | 41 |
| Sausage | Lower (P20) | **-** | 0 | 0 | 1 |
|  | Higher (P80) | - | 2 | 6 | 5 |
| Ham | Lower (P20) | 1 | 2 | 1 | 3 |
|  | Higher (P80) | 11 | 20 | 22 | 20 |
| Meat | Lower (P20) | 55 | 72 | 70 | 78 |
|  | Higher (P80) | 93 | 92 | 109 | 106 |
| Egg | Lower (P20) | 2 | 2 | 0 | 0 |
|  | Intermediate/Higher (P40) | 18 | 17 | 18 | 24 |
| Fish & Seafood | Lower (P20) | 24 | 22 | 23 | 23 |
|  | Intermediate/Higher (P40) | 64 | 79 | 94 | 79 |
| Chocolate milk | Lower (P20) | **-** | 0 | 0 | 0 |
|  | Higher (P80) | - | 84 | 116 | 109 |
| Yoghurt | Lower (P20) | 86 | 37 | 41 | 6 |
|  | Higher (P80) | 224 | 238 | 121 | 113 |
| Milk | Lower (P20) | 367 | 143 | 72 | 52 |
|  | Higher (P80) | 487 | 476 | 259 | 249 |
| Margarine | Lower (P20) | - | - | 0 | 0 |
|  | Higher (P80) | **-** | - | 3 | 3 |
| Cheese | Lower (P20) | 0 | 0 | 0 | 1 |
|  | Higher (P80) | 15 | 20 | 16 | 24 |
| Vegetables | Lower (P20) | 46 | 56 | 41 | 26 |
|  | Higher (P80) | 128 | 134 | 133 | 121 |
| Vegetable soup | Lower (P20) | 178 | 208 | 116 | 11 |
|  | Intermediate/Higher (P40) | 477 | 481 | 379 | 198 |
| Pulses | Lower (P20) | - | **-** | 0 | 0 |
|  | Higher (P80) | **-** | - | 26 | 24 |
| Fruits | Lower (P20) | 71 | 81 | 74 | 29 |
|  | Higher (P80) | 238 | 254 | 237 | 183 |
| Natural fruit juice | Lower (P20) | - | 0 | 0 | 0 |
|  | Higher (P80) | **-** | 12 | 22 | 44 |
| Nectar | Lower (P20) | 0 | 0 | 0 | 0 |
|  | Higher (P80) | 22 | 56 | 74 | 62 |
| Soft drinks (cola) | Lower (P20) | 0 | 0 | 0 | 0 |
|  | Higher (P80) | 6 | 16 | 31 | 28 |
| Soft drinks (iced-tea) | Lower (P20) | 0 | 0 | 0 | 0 |
|  | Higher (P80) | 51 | 127 | 90 | 99 |
| Soft drinks (other) | Lower (P20) | 0 | 0 | 0 | 0 |
|  | Higher (P80) | 69 | 84 | 106 | 104 |
| Table sugar | Lower (P20) | 0 | 0 | 0 | 0 |
|  | Higher (P80) | 3 | 1 | 2 | 2 |
| Coffee | Lower (P20) | 0 | 0 | 0 | 0 |
|  | Higher (P80) | 3 | 2 | 1 | 5 |
| Tea | Lower (P20) | 0 | 0 | 0 | - |
|  | Higher (P80) | 11 | 14 | 19 | - |

^a^ Lower and Higher groups represent the 1st and 5th quintiles of daily consumption, respectively. The intermediate group (2nd–4th quintiles) is not shown, as it reflects values between these groups.

Some items were not assessed at all evaluation follow-ups.

Supplemental Material 4. Conditional Item Response Probabilities for each food item by class (dietary pattern).

| **Item** |  | **Class 1**  **(Healthier)** | **Class 2**  **(EDF)** | **Class 3**  **(Lower Consumption)** |
| --- | --- | --- | --- | --- |
| Bread & toast | Lower | 0.34 | 0.35 | **0.50** |
|  | Intermediate | **0.62** | 0.60 | 0.48 |
|  | Higher | 0.04 | **0.05** | 0.02 |
| Plain biscuits & crackers | Lower | 0.28 | 0.32 | **0.45** |
|  | Intermediate | **0.66** | 0.57 | 0.43 |
|  | Higher | 0.06 | 0.11 | **0.12** |
| Cookies & biscuits | Lower | **0.57** | 0.28 | 0.52 |
|  | Intermediate | 0.34 | **0.47** | 0.30 |
|  | Higher | 0.09 | **0.26** | 0.18 |
| Cakes | Lower | **0.56** | 0.28 | 0.48 |
|  | Intermediate | 0.35 | **0.43** | 0.41 |
|  | Higher | 0.09 | **0.28** | 0.11 |
| Chocolate | Lower | 0.35 | 0.18 | **0.38** |
|  | Intermediate | 0.58 | **0.65** | 0.48 |
|  | Higher | 0.07 | **0.18** | 0.14 |
| Ice cream | Lower | 0.32 | 0.22 | **0.41** |
|  | Intermediate | **0.58** | 0.54 | 0.47 |
|  | Higher | 0.10 | **0.24** | 0.12 |
| Candies | Lower | **0.77** | 0.38 | 0.69 |
|  | Intermediate | 0.18 | **0.39** | 0.21 |
|  | Higher | 0.05 | **0.23** | 0.10 |
| Salty pastry | Lower | 0.40 | 0.16 | **0.41** |
|  | Intermediate | 0.54 | **0.61** | 0.53 |
|  | Higher | 0.05 | **0.23** | 0.05 |
| Salty snacks | Lower | **0.82** | 0.35 | 0.70 |
|  | Intermediate | 0.16 | **0.44** | 0.23 |
|  | Higher | 0.02 | **0.21** | 0.06 |
| Pizza & burger | Lower | **0.57** | 0.22 | 0.43 |
|  | Intermediate | 0.42 | **0.66** | 0.50 |
|  | Higher | 0.01 | **0.12** | 0.07 |
| Sausage | Lower | **0.72** | 0.28 | 0.55 |
|  | Intermediate | 0.24 | **0.44** | 0.33 |
|  | Higher | 0.05 | **0.28** | 0.12 |
| Ham | Lower | 0.29 | 0.14 | **0.32** |
|  | Intermediate | 0.59 | **0.68** | 0.57 |
|  | Higher | 0.12 | **0.18** | 0.11 |
| Meat | Lower | 0.28 | 0.37 | **0.45** |
|  | Intermediate | **0.69** | 0.54 | 0.42 |
|  | Higher | 0.02 | 0.10 | **0.14** |
| Egg | Lower | 0.36 | 0.40 | **0.69** |
|  | Intermediate | **0.64** | 0.60 | 0.31 |
|  | Higher | - | - | - |
| Fish & Seafood | Lower | 0.31 | 0.64 | **0.74** |
|  | Intermediate | **0.69** | 0.36 | 0.26 |
|  | Higher | - | - | - |
| Chocolate milk | Lower | **0.44** | 0.30 | 0.37 |
|  | Intermediate | 0.49 | **0.54** | 0.47 |
|  | Higher | 0.07 | **0.16** | **0.16** |
| Yoghurt | Lower | 0.33 | 0.27 | **0.47** |
|  | Intermediate | 0.58 | **0.62** | 0.48 |
|  | Higher | 0.09 | **0.10** | 0.04 |
| Milk | Lower | 0.19 | 0.22 | **0.25** |
|  | Intermediate | **0.68** | **0.68** | 0.67 |
|  | Higher | **0.13** | 0.10 | 0.08 |
| Margarine | Lower | 0.73 | 0.63 | **0.76** |
|  | Intermediate | 0.15 | **0.21** | 0.13 |
|  | Higher | 0.12 | **0.16** | 0.11 |
| Cheese | Lower | 0.20 | 0.21 | **0.33** |
|  | Intermediate | 0.67 | **0.67** | 0.62 |
|  | Higher | **0.13** | 0.11 | 0.05 |
| Vegetables | Lower | 0.11 | 0.25 | **0.40** |
|  | Intermediate | **0.65** | **0.65** | 0.58 |
|  | Higher | **0.24** | 0.10 | 0.03 |
| Vegetable soup | Lower | 0.15 | **0.40** | **0.40** |
|  | Intermediate | **0.85** | 0.60 | 0.60 |
|  | Higher | - | - | - |
| Pulses | Lower | 0.29 | 0.42 | **0.69** |
|  | Intermediate | **0.47** | 0.42 | 0.25 |
|  | Higher | **0.24** | 0.16 | 0.06 |
| Fruits | Lower | 0.09 | 0.38 | **0.53** |
|  | Intermediate | **0.82** | 0.57 | 0.46 |
|  | Higher | **0.09** | 0.05 | 0.02 |
| Natural fruit juice | Lower | 0.42 | 0.50 | **0.76** |
|  | Intermediate | **0.43** | 0.37 | 0.20 |
|  | Higher | **0.16** | 0.13 | 0.04 |
| Nectar | Lower | 0.48 | 0.32 | **0.66** |
|  | Intermediate | 0.42 | **0.47** | 0.24 |
|  | Higher | 0.10 | **0.21** | 0.09 |
| Soft drinks (cola) | Lower | 0.63 | 0.29 | **0.62** |
|  | Intermediate | 0.35 | **0.52** | 0.32 |
|  | Higher | 0.02 | **0.19** | 0.06 |
| Soft drinks (iced tea) | Lower | **0.59** | 0.17 | 0.57 |
|  | Intermediate | 0.36 | **0.55** | 0.27 |
|  | Higher | 0.05 | **0.27** | 0.16 |
| Soft drinks (other) | Lower | **0.58** | 0.16 | 0.57 |
|  | Intermediate | 0.36 | **0.54** | 0.29 |
|  | Higher | 0.06 | **0.30** | 0.13 |
| Table sugar | Lower | **0.78** | 0.57 | 0.77 |
|  | Intermediate | 0.12 | **0.18** | 0.10 |
|  | Higher | 0.10 | **0.25** | 0.13 |
| Coffee | Lower | **0.87** | 0.65 | 0.79 |
|  | Intermediate | 0.05 | **0.12** | 0.10 |
|  | Higher | 0.09 | **0.23** | 0.11 |
| Tea | Lower | 0.58 | 0.59 | **0.74** |
|  | Intermediate | **0.25** | 0.22 | 0.17 |
|  | Higher | 0.17 | **0.20** | 0.09 |

Patterns identified with latent class analysis (n=5247). Analysis included dietary data collected through a Food Frequency Questionnaire at 4, 7, 10, and 13 years of age. Daily consumption was categorised into three groups: lower (1^st^ quintile), intermediate (2^nd^–4^th^ quintiles), and higher (5^th^ quintile). For fish/seafood, eggs, and soup, the intermediate and higher consumption categories were combined. The highest probability for the category is in bold.

Supplemental Material 5. Associations between appetitive trait trajectory profiles and two outcomes: the dietary patterns at the older age (age 13) and using the longitudinal trajectories from 4 to 13 years.

|  | | Dietary Patterns ^a^ | | | | |
| --- | --- | --- | --- | --- | --- | --- |
|  |  | **Age 13** | | | **Trajectories from ages 4 to 13** | |
| Appetitive trait trajectory profiles ^b^  (Moderate as reference) | | Healthier | Energy-Dense Foods | Lower Consumption | Predominantly  Healthier | Predominantly  Energy-Dense Foods |
|  |  | OR (95% CI) | OR (95% CI) | OR (95% CI) | OR (95% CI) | OR (95% CI) |
| Small appetite but increasing | Crude | 1 | **1.76 (1.30,2.40)** | **2.23 (1.56,3.20)** | 1 | **1.74 (1.38,2.21)** |
|  | Adjusted | 1 | **1.46 (1.06,2.02)** | **2.05 (1.42,2.97)** | 1 | **1.54 (1.20,1.99)** |
| Small to moderate appetite | Crude | 1 | **0.79 (0.66,0.95)** | 0.95 (0.76,1.20) | 1 | **0.71 (0.61,0.82)** |
|  | Adjusted | 1 | **0.77 (0.64,0.93)** | 0.95 (0.75,1.20) | 1 | **0.69 (0.59,0.82)** |
| Avid appetite | Crude | 1 | **0.67 (0.53,0.86)** | 1.08 (0.81,1.43) | 1 | **0.80 (0.66,0.97)** |
|  | Adjusted | 1 | **0.58 (0.45,0.74)** | 1.00 (0.75,1.35) | 1 | **0.66 (0.54,0.81)** |
| Increasing appetite | Crude | 1 | 1.02 (0.82,1.27) | **1.32 (1.01,1.72)** | 1 | **1.23 (1.03,1.48)** |
|  | Adjusted | 1 | 0.85 (0.68,1.07) | 1.20 (0.91,1.58) | 1 | 1.08 (0.89,1.32) |
| Smallest appetite | Crude | 1 | 0.98 (0.76,1.26) | **1.35 (1.00,1.83)** | 1 | 0.90 (0.74,1.10) |
|  | Adjusted | 1 | 1.06 (0.81,1.37) | **1.44(1.05,1.96)** | 1 | 1.03 (0.83,1.28) |

OR: Odds ratio. CI: confidence interval; ref: reference category. ^a^ Dietary Patterns identified with latent class analysis, using dietary data collected at 4, 7, 10, and 13 years of age. ^b^ Appetitive trait profiles were driven with data from the Children’s Eating Behaviour Questionnaire at 7,10, and 13 years of age [1]. ^c^ Models adjusted for child’s sex, physical activity at age 4, mothers’ education, age, and household income.N is 4232 at age 13, and 5040 for the trajectories.

Supplemental Material 6. Associations between appetitive trait trajectory profiles and two outcomes: the dietary patterns at the older age (age 13) and using the longitudinal trajectories from 4 to 13 years only including participants with plausible dietary reports*.

|  | |  | Dietary Patterns ^a^ | | | |
| --- | --- | --- | --- | --- | --- | --- |
|  |  | **Age 13** | | | **Trajectories from ages 4 to 13** | |
| Appetitive trait trajectory profiles ^b^  (Moderate as reference) | | Healthier | Energy-Dense Foods | Lower Consumption | Predominantly Healthier | Predominantly Energy-Dense Foods |
|  |  | OR (95% CI) | OR (95% CI) | OR (95% CI) | OR (95% CI) | OR (95% CI) |
| Small appetite but increasing | Crude | 1 | **1.74 (1.22,2.49)** | **2.60 (1.70,3.98)** | 1 | **1.79 (1.40,2.30)** |
|  | Adjusted | 1 | **1.46 (1.01,2.11)** | **2.26 (1.47,3.48)** | 1 | **1.58 (1.19,2.11)** |
| Small to moderate appetite | Crude | 1 | **0.78 (0.63,0.95)** | 1.05 (0.80,1.38) | 1 | **0.72 (0.62,0.84)** |
|  | Adjusted | 1 | **0.73 (0.58,0.90)** | 1.00 (0.75,1.33) | 1 | **0.71 (0.60,0.86)** |
| Avid appetite | Crude | 1 | 0.77 (0.58,1.02) | 1.23 (0.87,1.75) | 1 | 0.84 (0.69,1.03) |
|  | Adjusted | 1 | **0.66 (0.49,0.88)** | 1.08 (0.75,1.54) | 1 | **0.66 (0.52,0.84)** |
| Increasing appetite | Crude | 1 | 1.09 (0.85,1.39) | **1.55 (1.13,2.13)** | 1 | **1.27 (1.05,1.53)** |
|  | Adjusted | 1 | 0.90 (0.66,1.16) | 1.36 (0.99,1.87) | 1 | 1.16 (0.93,1.45) |
| Smallest appetite | Crude | 1 | 0.85 (0.64,1.12) | **1.51 (1.07,2.13)** | 1 | 0.88 (0.72,1.09) |
|  | Adjusted | 1 | 0.89 (0.66,1.19) | **1.58 (1.12,2.24)** | 1 | 0.98 (0.77,1.24) |

OR: Odds ratio. CI: confidence interval; ref: reference category. ^a^ Dietary Patterns identified with latent class analysis, using dietary data collected at 4, 7, 10, and 13 years of age. ^b^ Appetitive trait profiles were driven with data from the Children’s Eating Behaviour Questionnaire at 7,10, and 13 years of age [1].^c^ Models adjusted for child’s sex, physical activity at age 4, mothers’ education, age, and household income. ^d^

* Misreporting was estimated by calculating the ratio between the reported total energy intake (TEI) and the estimated energy requirement (EER) [2,3]. The EER was calculated using validated sex- and age-specific equations, considering the physical activity category (low active), exact age, and objectively measured weight and height [4]. Participants were categorised as plausible reporters, under-reporters, or over-reporters of TEI using the ±1 SD cut-off for the ratio TEI/EER [2,3]. At age 13, 3153 were included in this analysis, being classified as plausible reports. For the trajectories, we considered participants with a plausible report for most follow-ups (n=4728).

**References**

1. Costa A, Pereira R, Severo M, Hetherington MM, Oliveira A. Appetitive traits from childhood to adolescence: Analysis of their stability, derivation of trajectory profiles, and associated characteristics. Appetite. 2024;193:107149. doi:10.1016/J.APPET.2023.107149
2. Huang TTK, Roberts SB, Howarth NC, McCrory MA. Effect of Screening Out Implausible Energy Intake Reports on Relationships between Diet and BMI. Obes Res. 2005;13(7):1205-1217. doi:10.1038/OBY.2005.143
3. Leech RM, Worsley A, Timperio A, McNaughton SA. The role of energy intake and energy misreporting in the associations between eating patterns and adiposity. Eur J Clin Nutr. 2018;72(1):142-147. doi:10.1038/EJCN.2017.90
4. National Academies of Sciences E and M. Dietary Reference Intakes for Energy. Dietary Reference Intakes for Energy. Published online January 17, 2023:1-526. doi:10.17226/26818
